# Challenges in Treating Statin-Associated Necrotizing Myopathy

**DOI:** 10.1155/2021/8810754

**Published:** 2021-02-24

**Authors:** Patrick Webster, Nicholas Wiemer, Abdalhamid Al Harash, Cody Marshall, Nazia Khatoon, Michael Lucke

**Affiliations:** ^1^Department of Internal Medicine, Allegheny Health Network, Pittsburgh, USA; ^2^Department of Rheumatology, Allegheny Health Network, Pittsburgh, USA; ^3^Department of Pathology, Allegheny Health Network, Pittsburgh, USA

## Abstract

Myalgia and mild elevation in muscle enzymes are common side effects of statin therapy. While these symptoms are generally self-limited, in rare cases, statin use is associated with an immune-mediated necrotizing myopathy caused by development of autoantibodies against HMG-CoA reductase. The primary presenting symptom of this condition is progressive symmetric proximal weakness that does not abate or worsens even after cessation of statin therapy and is associated with markedly elevated creatine kinase (CK) levels. To date, no randomized controlled trials have been conducted to identify the most effective treatment for statin-associated autoimmune myopathy. Treatment recommendations involve a combination of steroids and immunosuppressive drugs. This single-center case series highlights the clinicopathologic features diagnostic for statin-associated autoimmune myopathy as well as treatment challenges for the patient population. The series highlights a range of potential presentations, from mildly symptomatic despite highly elevated CK, to severe muscle weakness including dysphagia. Multiple patients required several immunosuppressant medications as well as intravenous immunoglobulin (IVIG) to achieve disease control. In this case series, marked improvement was noted in several diabetic patients with IVIG.

## 1. Introduction

Statin therapy has been shown to reduce the incidence of cardiovascular disease and is used routinely throughout medical practice. A common side effect of statin use is myalgia and mild elevation in muscle enzymes. These symptoms can result in muscle damage, but this reaction is self-limiting and abates with cessation of statin [1]. However, in rare cases, statin use can result in statin-associated autoimmune myopathy caused by the development of autoantibodies against HMG-CoA reductase [2]. Findings of a positive anti-HMG-CoA reductase (anti-HMGCR) antibody in the appropriate clinical context are highly suggestive of the diagnosis, which is further established with muscle biopsy pathology demonstrating a necrotizing myopathy with muscle-cell necrosis and regeneration [3]. Patients usually present clinically with progressive symmetric proximal weakness that may persist or worsen even after statin therapy is discontinued [2–6]. To date, no randomized controlled trials have been conducted to identify the most effective treatment for statin-associated autoimmune myopathy, but first-line treatment is typically a combination of steroids and immunosuppressive drugs [4, 5, 7]. In addition to immunosuppressive treatment, compelling data highlights the treatment benefits of IVIG [4, 6, 8].

We report a single-center case series of seven patients with statin-associated autoimmune myopathy, highlighting the clinical heterogeneity in this disease as well as variable and outcomes associated with treatment via steroids, immunosuppression, and IVIG.

## 2. Case Description 1

A 71-year-old female with hypertension and hyperlipidemia treated with atorvastatin 40 mg presented with 6 months of progressive proximal muscle weakness and myalgia in the upper and lower extremities with a creatine kinase (CK) level of 13,871 IU/L. Of note, 2 years prior to this presentation, she experienced myopathy with elevated CK levels of 7,609 IU/L. ANA, anti Jo-1, SSA, SSB, and RNP were negative. The patient's atorvastatin was discontinued.

**Figure 1:**
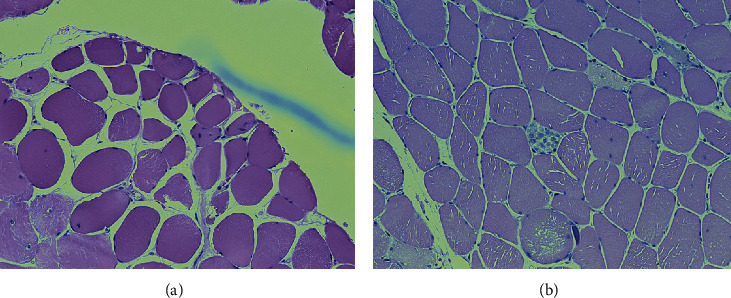
Regenerating (a) and degenerating (b) muscle fibers at 20*x* magnification.

Electromyography (EMG) showed abnormal insertional activity and positive fibrillation waves consistent with a myopathic pattern. Muscle biopsy revealed myonecrosis without an inflammatory infiltrate (Figure 1). The anti-HMG-CoA reductase (anti-HMGCR) antibody level was >200 U/ml.

The patient was initiated on prednisone 60 mg/day. Proximal weakness improved, and CK decreased to 6,366 IU/L. Despite medication adherence, her symptoms recurred 6 weeks later, at which time CK rose to 12,000 IU/L, prompting treatment with 1 g of intravenous methylprednisolone daily for 3 days. She was started on azathioprine 150 mg/day with a prednisone taper. The CK level downtrended to 311 IU/L over 10 months. The patient saw resolution of symptoms with 5/5 muscle strength in all 4 extremities after 9 months of treatment.

## 3. Case Description 2

**Figure 2:**
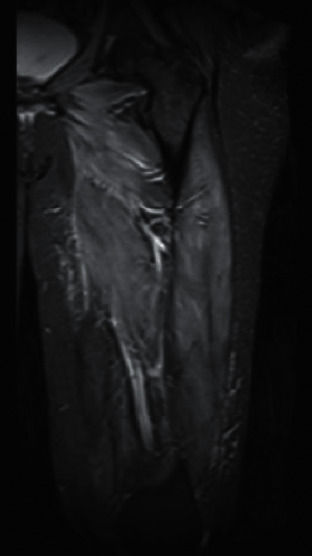
MRI and STIR weighted sequences of the left femur showing extensive intramuscular edema.

**Figure 3:**
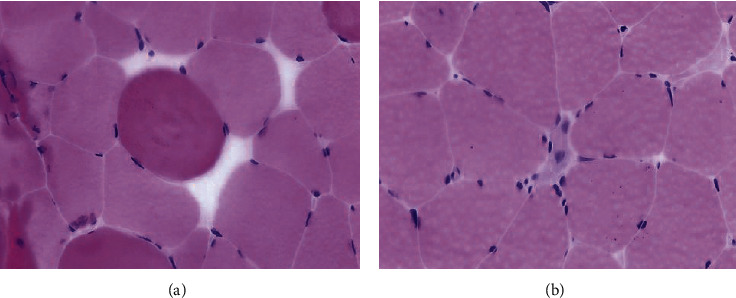
Degenerating (a) and regenerating (b) muscle fibers at 400*x* magnification.

A 54-year-old female presented with progressive muscle weakness over 2 months with impaired ambulation and multiple falls. She had a medical history of diabetes, hypertension, and hyperlipidemia treated with atorvastatin 80 mg. Labs were significant for a CK level of 24,300 IU/L. MRI of the left thigh showed extensive intramuscular edema and enhancement throughout the musculature (Figure 2). Muscle biopsy showed necrotizing myopathy with areas of degenerating and regenerating muscle fibers (Figure 3). Anti-HMGCR antibody was positive with a level of >200 U/ml. ESR, CRP, anti Jo-1, SSA, SSB, and RNP were within normal limits. Atorvastatin was changed to niacin, and the patient was started on prednisone 60 mg. The CK level decreased to 6,819 IU/L. Mycophenolate mofetil (MMF) 1000 mg twice daily was added, with minimal symptomatic relief following 1 month of therapy. IVIG 2 g/kg monthly was given for 1 year. Following 4 cycles of IVIG, the CK level continued to decrease to 1,610 IU/L and the patient's symptoms resolved. She was successfully tapered off steroids after a total of 5 months.

## 4. Case Description 3

A 64-year-old female with diabetes, hypertension, and hyperlipidemia presented with persistent lower extremity pain and weakness over 12 months. 6 months prior to presentation, atorvastatin was replaced with simvastatin due to suspicion of statin-associated myalgia.

Labs were significant for a CK level of 14,000 IU/L. ANA, SSA, SSB, and anti Jo-1 were negative. EMG showed increased insertional activity with positive fibrillation waves in the proximal upper and lower extremities and thoracic paraspinal muscles. Muscle biopsy showed necrosis with features of degeneration and regeneration without inflammation. Anti-HMGCR antibody was elevated at 167 U/ml.

Prednisone and MTX were started, and due to persistent symptoms over 4 months, azathioprine 100 mg/day was added. Over the following 8 months, weakness progressed and myalgias persisted, prompting a change to rituximab 1 g, 2 weeks apart. Following the rituximab infusions, she was maintained on MMF 2 g and achieved remission over 4 years. Symptoms then flared with a CK level of 1,800 IU/L. Despite retreatment with rituximab 1 g, CK remained >1000 IU/L and proximal muscle weakness worsened. She was started on IVIG 2 g/kg every 4 weeks while continuing 2 g MMF twice daily. She experienced symptomatic improvement, and CK dropped to 600 IU/L. She subsequently achieved remission on IVIG 1 g/kg every 4 weeks and MMF 2 g twice daily.

## 5. Case Description 4

A 76-year-old male with a past medical history of heart failure, coronary artery disease, hyperlipidemia, and diabetes presented with 6 weeks of bilateral upper and lower proximal muscle weakness. 2 years prior, he was started on 80 mg atorvastatin. A CK level of 6,333 IU/L prompted discontinuation of atorvastatin. Over the next 4 weeks, symptoms progressed including development of dysphagia. EMG showed abnormal insertional activity in the form of positive waves and fibrillation potentials. Muscle biopsy showed nonspecific degenerating and regenerating muscle fibers without inflammation. Anti-HMGCR antibody was elevated to 131 U/ml. ANA, anti-Smith, anti-dsDNA, ANCA, SSA, SSB, and RNP were negative. The patient was started on prednisone, MTX, and IVIG 2 g/kg monthly. The patient's dysphagia resolved, ambulation improved, and CK normalized.

The patient was started on evolocumab for hyperlipidemia. He maintained on MTX 25 mg/week and IVIG 2 g/kg per month with a prednisone taper. IVIG was stopped with clinical improvement; however, the patient flared after withdrawal of this therapy. IVIG was restarted at 2 g/kg a month, and azathioprine was started. With ongoing symptoms, azathioprine was changed to MMF. While on treatment, he developed a large right exudative pleural effusion with near-complete collapse of the right lung. Further evaluation with PET/CT did not show active myositis or evidence of malignancy, and the patient was maintained on IVIG monotherapy.

## 6. Case Description 5

A 66-year-old male with hypertension and hyperlipidemia on atorvastatin 80 mg for 2 years presented with proximal leg weakness over 10 months with 4/5 strength on examination. The CK level was 8,790 IU/L. Atorvastatin was discontinued, but symptoms worsened. On physical exam, the patient had 4/5 strength in the proximal upper and lower extremities. EMG showed fibrillation potentials and increased recruitment in the upper and lower extremities with truncal involvement. A muscle biopsy showed rare nonspecific atrophic fibers with no inflammation or necrosis. The anti-HMGCR antibody level was >200 U/ml. ANA, anti-centromere, SSA, and SSB were negative. The patient declined immunosuppression therapy and, despite persistent CK elevations ranging from 2,500 to 5,000 IU/L, continued to have only mild weakness without interruption in daily activities.

## 7. Case Description 6

A 69-year-old male with a past medical history of antiphospholipid syndrome, diabetes, hypertension, and hyperlipidemia, on atorvastatin 20 mg for four years, was hospitalized with acute hypoxic respiratory failure. The patient was found to have 3/5 hip flexor strength and reported having trouble walking for 3 months prior to admission. The patient had stopped atorvastatin 2 months prior to presentation. CK was 9,770 IU/L. EMG demonstrated a sensory and motor peripheral neuropathy. MRI showed increased T2 signal of the iliopsoas, gluteal, adductor, quadriceps, and semimembranosus muscles bilaterally. Muscle biopsy revealed necrotizing myopathy. Anti-HMGCR antibody was >200 U/mL. ANA, ANCA, anti Jo-1, CCP, PM SCL, and Lyme titers were negative. He was also found to have silent aspiration on modified barium swallow (MBS). His oxygenation improved, and he was discharged on prednisone 40 mg/day.

After discharge, the patient's weakness worsened and prednisone was increased to 80 mg/day. He was started on azathioprine 200 mg/day and IVIG 700 mg/kg administered daily for 3 days per month with marked clinical improvement but persistent CK elevation after 4 months. He was maintained on IVIG 2 g/kg monthly with azathioprine 50 mg daily.

## 8. Case Description 7

A 72-year-old male with a past medical history of diabetes, atrial fibrillation, hypertension, and hypercholesterolemia presented with 4/5 proximal muscle weakness on exam and CK of 7,802 IU/L. He had been treated with atorvastatin for the last 8 years. Although he had difficulty rising from a seated position, he reported that through a self-directed strengthening regimen, his strength had improved by 50%.

Muscle biopsy confirmed a necrotizing myositis. The anti-HMGCR antibody level was 192 U/mL. Mi-2, PL-7, PL-12, Ku, EJ, OJ, anti Jo-1, and SRP were negative. The patient declined immunosuppressive treatment and had persistent 4/5 strength in his lower extremities and improved but persistent CK elevation at 2,419 IU/L. He continued to decline treatment as he was feeling well with exercising regularly.

## 9. Discussion

Statin-associated autoimmune myopathy is estimated to occur in approximately 2-3 per 100,000 patients [9]. The primary presenting symptom is symmetrical proximal weakness, with clinicopathologic features including a marked increase of CK with the presence of autoantibodies against HMG-CoA reductase. All the patients encountered in this series were referred to our center with these hallmark diagnostic features (Table 1). While the etiopathogenesis of statin-associated autoimmune myopathy remains obscure, Basharat et al. reported that anti-HMG-CoA reductase myopathy is more common in patients with type 2 diabetes [10]. 5/7 patients in this cohort presented with a prior diagnosis of diabetes.

To date, no randomized controlled trials have been conducted to identify the most effective treatment for statin-associated autoimmune myopathy. Clinical reports have shown that steroids and immunosuppressive agents can improve symptoms [4, 5, 11]. Frequently prescribed therapies include prednisone with azathioprine, methotrexate, or mycophenolate mofetil [4, 8, 12]. Moreover, nearly half of the patients described in the literature were treated with three medications, most commonly with glucocorticoids, IVIG, and an additional immunosuppressant [12]. Our patient outcomes support the need for early immunosuppression in most patients. Although IVIG is often reserved for patients who have not benefitted from either steroid or oral immunosuppressive therapy, several suggestive studies have shown that IVIG could be used as a first-line monotherapy in select patients, particularly those with type 2 diabetes [3, 6]. Indeed, 4 of 5 patients with diabetes in our series achieved marked improvement after initiation of IVIG, suggesting a potential earlier role for this therapy in diabetic patients.

It remains unclear why certain patients with statin-associated autoimmune myopathy and anti-HMGCR antibodies see a spontaneous resolution of symptoms while the majority experience symptoms even after statin discontinuation. Patients 5 and 7 offer examples of this phenomenon. Both presented with mild cases of myopathy, and their symptoms and CK levels improved but remained elevated with statin cessation in the absence of immunosuppressive treatment. It has been postulated that although findings of muscle destruction persist as demonstrated by high CK levels, muscle regeneration may outpace destruction with resulting stability of symptoms [3]. Patient 7 implemented an early strengthening training, which in theory may assist with muscle regeneration.

Rechallenging with statin therapy may be considered in patients with mild myopathy or an isolated elevation in CK, but alternative therapies are recommended in cases of statin-associated autoimmune myopathy. In this series, one patient was treated with the monoclonal antibody evolocumab without worsening of the disease. Another patient was treated with niacin successfully.

## 10. Conclusion

Statin-associated autoimmune myopathy is a rare complication of statin use that has potentially debilitating consequences. Treatment consists of prompt discontinuation of statins and early immunosuppression. In this case series, marked improvement was noted in several diabetic patients with IVIG. This finding supports the consideration of early initiation of IVIG in diabetic patients with highly active statin-associated autoimmune myopathy, potentially as a first-line agent.

## Figures and Tables

**Table 1:** Summary of patients.

Patient	Age (years)	Sex	CK level (IU/L)	Anti-HMGCR antibody level (U/mL)	Primary biopsy findings	Statin	Therapy
1	71	Female	13,871	>200	Degenerating and regenerating muscle fibers without inflammation	Atorvastatin	Prednisone, methylprednisolone, AZA
2	54	Female	24,300	>200	Many regenerating and occasional necrotic muscle fibers with mild, patchy inflammation	Atorvastatin	Prednisone, MMF, IVIG
3	64	Female	14,000	167	Necrotic muscle fibers	Atorvastatin, simvastatin	Prednisone, MTX, AZA, rituximab, MMF, IVIG
4	76	Male	6,333	131	Numerous regenerating, degenerating, and atrophic muscle fibers without inflammation	Atorvastatin	Prednisone, MTX, IVIG, AZA, MMF
5	66	Male	8,790	>200	Few, atrophic muscle fibers	Atorvastatin	None
6	69	Male	9,770	>200	Degenerating and regenerating muscle fibers without signs of inflammation	Atorvastatin	Prednisone, AZA, IVIG
7	72	Male	7,802	192	Numerous degenerating and regenerating fibers without inflammation	Atorvastatin	None

HMGCR = HMG-CoA reductase, AZA = azathioprine, IVIG = intravenous immunoglobulin, MMF = mycophenolate mofetil, and MTX = methotrexate.

## Data Availability

The research data used to support the findings of this case series are included within the article.
